# Exploring the “poppy seed defense” in oral fluid: detection of opioids following poppy seed consumption

**DOI:** 10.1093/jat/bkaf088

**Published:** 2025-09-23

**Authors:** Lena Midtlyng, Gudrun Høiseth, Rafika Rahho, Cecilie Hasselø Thaulow

**Affiliations:** Department of Forensic Sciences, Division of Laboratory Medicine, Oslo University Hospital, 0424 Oslo, Norway; Department of Forensic Sciences, Division of Laboratory Medicine, Oslo University Hospital, 0424 Oslo, Norway; Institute of Clinical Medicine, Medical Faculty, University of Oslo, 0318 Oslo, Norway; Center for Psychopharmacology, Diakonhjemmet Hospital, 0319 Oslo, Norway; Department of Forensic Sciences, Division of Laboratory Medicine, Oslo University Hospital, 0424 Oslo, Norway; Department of Forensic Sciences, Division of Laboratory Medicine, Oslo University Hospital, 0424 Oslo, Norway

**Keywords:** Oral fluid, Drug testing, Poppy seeds, Opioids, Morphine

## Abstract

The “poppy seed defense”—a claim that a positive opioid test result is due to ingestion of poppy seeds—is occasionally encountered in forensic toxicology. The matter has been thoroughly investigated in urine but is less researched in oral fluid. We therefore aimed to perform an experimental study to explore whether consumption of commercially available poppy seeds would lead to detection of opioids in oral fluid. Additionally, we aimed to relate our findings to routine cases. Ten volunteers consumed either five crispbreads containing a small amount of poppy seeds, or 30 g of raw poppy seeds with a low opioid content (3.0 mg/kg morphine and 0.9 mg/kg codeine). Oral fluid samples were collected 0.5 and 2 hours after consumption. Additionally, a urine sample was collected 2 hours after consumption. Following ingestion of raw seeds, morphine was detected (estimated neat oral fluid concentrations 1.4–5.6 ng/mL) in all oral fluid samples 0.5 hours after consumption and in one (2.4 ng/mL) of five oral fluid samples after 2 hours. Codeine was detected (0.8–1.1 ng/mL) in three of five oral fluid samples 0.5 hours after consumption, but in none after 2 hours. Following ingestion of crispbreads, morphine or codeine was not detected in oral fluid, but opioids/-glucuronides were detected in three of five urine samples. When comparing our results with routine cases, we found that 14% of routine cases had morphine concentrations in oral fluid samples lower or similar to those seen after ingestion of raw seeds in our experimental study. In conclusion, we found that consumption of raw seeds led to detection of opioids in oral fluid, but the detection window appeared to be short. Comparison with routine cases indicated that the poppy seed defense may be a challenge when interpreting oral fluid results, particularly when low cut-off levels are applied.

## Introduction

Detection of opioids in biological samples is occasionally attributed to consumption of poppy seeds, known as “the poppy seed defense.” Poppy seeds do not naturally contain opioids but may become contaminated with opioid alkaloids from other parts of the opium poppy plant [1]. The opium poppy (*Papaver ­somniferum* L.) may contain several different opioid alkaloids [2], with morphine and codeine being the most relevant to drug testing. Analyses show that the morphine content in poppy seeds can vary considerably, with concentrations ranging from negligible levels to several hundred mg/kg [1, 3]. Poppy seeds are commonly used in a variety of food products, such as baked goods and warm dishes, or raw in salad dressings or muesli. When detecting morphine or codeine in biological samples, it may be challenging to differentiate an intake of poppy seeds from intake of opioid drugs.

Drug analyses in oral fluid, rather than urine, have become increasingly common due to the straightforward sampling method. Oral fluid testing is also employed in contexts where the results may have legal consequences, such as in workplace drug testing and correctional facilities.

While several previous studies have investigated opioid detection in urine following poppy seed ingestion [4–8], data on opioid detection in oral fluid is limited. Although some studies have shown detection of opioids in oral fluid after consumption of poppy seeds, it is uncertain how the results apply to routine cases, as the opioid content consumed in the studies was either high [9–11] or unknown [12–14]. Additionally, the treatment of the seeds prior to ingestion, as well as the oral fluid sampling devices, analytical methods and cut-off levels, varied across the studies.

In December 2021, the European Union (EU) implemented a regulation for maximum opium alkaloid levels in foodstuffs. The maximum level for opium alkaloid content was set to 20 mg/kg of morphine equivalents for poppy seeds and 1.50 mg/kg for bakery products containing poppy seeds. Codeine is converted to morphine equivalents by multiplying with a factor of 0.2 [15]. Introduction of these maximum levels should lower the concentrations of morphine and codeine in poppy seeds sold on the European market and thus reduce the risk of opioid detection in biological samples after consumption of poppy seeds.

Therefore, the aim of this study was to perform an experimental study to explore whether the consumption of raw poppy seeds and baked goods containing poppy seeds, commercially available in Europe, would result in the detection of opioids in oral fluid. Urine was also sampled for comparison. Additionally, we aimed to relate our experimental study findings to opioid results from routine cases.

## Materials and methods

### Experimental study

#### Participants

We recruited ten healthy adult volunteers for consumption of either raw poppy seeds or crispbreads containing poppy seeds. Exclusion criteria were the use of opioids that could lead to detection of morphine or codeine within two weeks prior to the study day, known allergies to any of the administered alimentary products, and pregnant or lactating women. Participants were randomized into two groups of five participants: a Raw seed group and a Crispbread group.

#### Materials

Five different brands of raw poppy seeds commercially available in Norway were purchased. The opioid content was analyzed, and the raw seed product with the highest morphine content (TRS Foods, Poppy seeds White, Asia’s finest) was used in the experimental study.

The crispbreads were of the brand Wasa frukost, a traditional Scandinavian dry bread. Five crispbreads contain approximately 3–4 g of poppy seeds in total, estimated from the product ingredient list. The opioid content of the poppy seeds from the crispbreads was not analyzed.

Oral fluid was collected with the Intercept^®^ i2 Oral Fluid Collection Device (OraSure Technologies, Bethlehem, PA, USA). The device’s pad is designed to collect 1 mL of saliva, as indicated by a color change in the volume adequacy indicator window. After sampling, the pad is placed into a vial containing 2 mL of preservative solution. A few hours after specimen collection, the pads were removed from the vials without prior centrifugation, and aliquots of 100 µL oral fluid-preservative solution mixture were transferred to separate 5 mL polypropylene tubes (Sarstedt AG, Rommelsdorf, Germany) and stored 1–5 days at 4°C until analysis.

Urine samples were collected in no additive urine collection containers and transferred to Vacuette^®^ 10 mL Z Urine No Additive tubes (Greiner Bio-One GmbH, Kremsmünster, Austria). Aliquots of 100 μL urine were transferred to separate 5 mL polypropylene tubes from Sarstedt AG and stored at 4°C for 1–6 days until analysis.

#### Study design

On the study day, after an overnight fast, participants provided an oral fluid sample prior to the ingestion of either raw poppy seeds or crispbreads.

Participants in the Raw seed group consumed 30 g of raw poppy seeds. Participants in the Crispbread group consumed five crispbreads with raspberry jam as topping, to facilitate consumption. All participants were allowed to drink water freely during the study day, including during the consumption of raw seeds and crispbreads, to ease the ingestion. We did not instruct participants to actively rinse their mouths after consumption.

Oral fluid samples were collected 0.5 and 2 hours after ingestion. Oral fluid sampling devices were retained in the mouth until the indicator window changed color, signifying adequate collection volume, or for a minimum duration of five minutes. Participants provided a urine sample 2 hours after ingestion. The participants fasted, apart from water, until the first oral fluid sample had been collected. They were then allowed to consume food, but asked to ensure that the food did not contain poppy seeds, until all biological samples were collected.

### Analytical methods

Sample preparations of 100 μL authentic sample, oral fluid-preservative solution mixture or urine, were automated using a Tecan Freedom Evo system from Tecan Group LTD (Männedorf, Switzerland) and analyzed by liquid chromatography-tandem mass spectrometry (LC–MS/MS). All analytical methods are routinely used in casework involving urine and oral fluid samples in our laboratory.

Results below the limit of quantification (LOQ) are reported as “not detected.” In the experimental study, the methods’ LOQ were used as the cut-off levels. Neat oral fluid concentrations were estimated based on an assumed oral fluid volume of 1 mL.

#### Oral fluid

The oral fluid samples were analyzed for morphine and codeine using a slightly modified previously published LC–MS/MS method [16]. Sample preparation was performed by 96-well supported liquid extraction (SLE), but by using 100 µL and not 200 µL sample volume. Analyses were performed in the oral fluid-preservative solution mixtures without measuring the oral fluid volume. LC–MS/MS analysis was performed on an Acquity ultra performance liquid chromatography (UPLC), coupled to a Xevo TQ-S MS/MS, all from Waters Corp. (Milford, MA, USA). Chromatographic separations were performed on an Acquity BEH C18 (2.1 × 50 mm, 1.7 μm particles) column with an Acquity BEH C18 precolumn (2.1 × 5 mm, 1.7 μm particles), both from Waters (Wexford, Ireland). The mobile phase consisted of 5 mM ammonium formate pH 10.2, and methanol. Chromatographic peaks were narrow and symmetrical. Analysis was performed with positive electrospray ionization (ESI^+^). The LOQ for both morphine and codeine was 0.09 ng/mL.

According to Valen et al., the linear range for morphine and codeine was 0.043–17.3 ng/mL and 0.045–18.0 ng/mL, respectively, the inter-assay precision and accuracy were within −10% to 9% for morphine and codeine, and recovery was between 71% and 75% [16]. The same study also found that matrix effects were within 82%–121% and internal standard corrected matrix effects were within 99%–101%.

Another study investigated drug recovery from the Intercept oral fluid collection device pad to evaluate the possibility of drug loss due to adsorption to the pad. They found no significant drug loss for codeine and morphine, and a recovery rate of 80% and 83%, respectively [17].

#### Urine

The urine samples were analyzed for morphine, codeine, morphine-3-glucuronide (M3G), morphine-6-glucuronide (M6G) and codeine-6-glucuronide (C6G). Stable isotope labeled internal standards were used for all compounds, except C6G. The sample preparation for urine was based on “dilute-and-shoot” approach and consisted of a simple dilution of 100 µL of urine with 900 µL methanol/type 1 water (1:1, v:v). LC–MS/MS analyses were performed on an Acquity UPLC coupled to a Xevo TQ tandem mass spectrometer from Waters Corp. Chromatographic separation was performed as described in a previous study on an Acquity BEH C18 (2.1 × 50 mm, 1.7 μm particles) column with a 5 mm precolumn with the same material, and mobile phase consisted of 5 mM ammonium formate pH 10.2, and methanol [18]. MS/MS analysis was performed with ESI^+^. The LOQ was 8.6 ng/mL for morphine, 18.0 ng/mL for codeine, 27.7 ng/mL for M3G and M6G, and 28.5 ng/mL for C6G.

Urine samples were screened for creatinine by immunoassay on an AU680 Clinical Chemistry Analyzer (Beckman Coulter Inc., Pasadena, CA, USA) using Creatinine-Detect Test reagent from DRI Thermo Fisher Scientific (Waltham, MA, USA). The LOQ for creatinine was 11.3 mg/L.

#### Poppy seeds analysis

The raw poppy seeds were manually ground using a mortar. To avoid contamination, the mortar was washed after each use. Approximately 20 mg of each type of ground seed batch was weighed into a 2 mL glass volumetric flask, and methanol was added to produce 2 mL of mixture. The flasks were placed into an ultrasonic bath for 10 minutes, and aliquots of 100 μL were transferred to separate 5 mL polypropylene tubes from Sarstedt AG and stored at 4°C until analysis. The samples were prepared and analyzed using the same analytical method as the urine samples, “dilute and shoot” LC–MS/MS.

### Routine cases

We explored anonymous results of oral fluid samples from the routine database at the Department of Forensic Sciences, Oslo University Hospital, Norway. Cases where morphine or codeine were detected at or above the laboratory’s cut-off levels (0.86 and 0.90 ng/mL for morphine and codeine, respectively) from July 2018 to December 2024, were included. Cases in which 6-acetylmorphine (6-AM) was detected, indicating heroin use, were excluded. All samples had been analyzed using the same analytical method as described in the experimental study.

### Data analysis

We used Microsoft Excel 2016, SPSS version 28.0-30.0 (IBM, Armonk, NY, USA) and GraphPad Prism (version 10.1.2, GraphPad Software LLC, Boston, MA, USA) for data handling and figure generation.

### Ethics

All participants in the experimental study provided written, informed consent. The experimental study was approved by the Data Protection Officer at Oslo University Hospital (reference number 22/27735). The Regional Committee for Medical and Health Research Ethics (REC) reviewed the study and determined that it does not fall within the scope of the Health Research Act.

We considered the amount of morphine equivalents ingested in the experimental study safe for health, traffic safety, and working conditions.

Only anonymized results from routine cases were retrieved from the database, according to the current data processing agreement between the Department of Forensic Sciences, Oslo University Hospital and the Norwegian Higher Prosecuting Authority.

## Results

### Experimental study

#### Study participants

The participants were 32 to 61 years of age, with a median age of 49 years. There were three women and two men in both study groups.

The ingestion of raw seeds and crispbreads was uncomplicated, and all participants completed the study protocol without major hesitation.

#### Poppy seeds

The analyzed batch of the seed product used in the study was found to contain 3.0 mg/kg morphine and 0.9 mg/kg codeine.

#### Oral fluid and urine results

Opioids were not detected in any of the oral fluid samples collected prior to the ingestion of poppy seeds.

**Figure 1.**
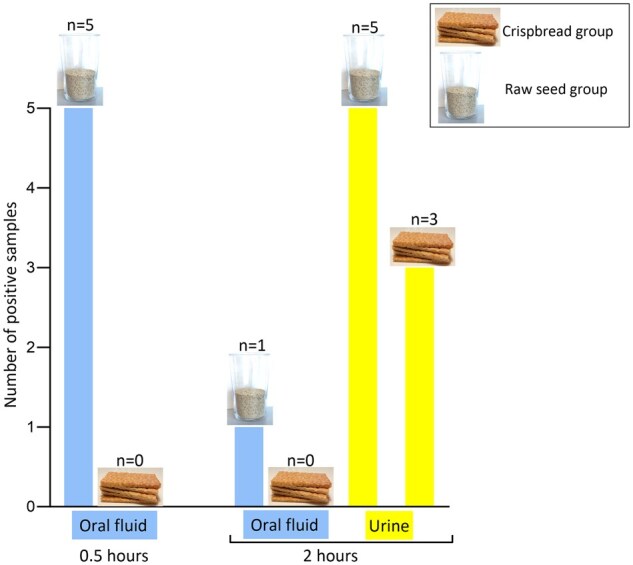
Number of positive samples (*n*, opioid or opioid glucuronide detected) per study group in oral fluid and urine 0.5 and 2 hours after consumption of either 30 g of raw poppy seeds or five crispbreads.

Table 1 shows the individual results from oral fluid and urine samples following the ingestion of raw seeds or crispbreads. Figure 1 illustrates the number of positive samples in oral fluid and urine for each study group and collection time point.

**Table 1.** The results from opioid analyses in oral fluid and urine for each of the ten study participants (#) after ingestion of either 30 g of raw poppy seeds or five crispbreads

	#	Oral fluid (ng/mL, estimated neat concentrations)				Urine (ng/mL, crea: mg/L)					
		0.5 h		2.0 h		2.0 h					
		M	C	M	C	M	M3G	M6G	C	C6G	Crea
Raw seed group	1	1.4	n.d.	n.d.	n.d.	n.d.	148	46.2	n.d.	n.d.	181
	2	3.3	1.1	n.d.	n.d.	28.5	337	111	n.d.	71.3	509
	3	2.5	n.d.	n.d.	n.d.	11.4	305	78.5	n.d.	33.3	373
	4	5.6	0.8	2.4	n.d.	n.d.	180	64.6	n.d.	42.8	170
	5	4.2	0.9	n.d.	n.d.	n.d.	111	27.7	n.d.	n.d.	238
Crispbread group	6	n.d.	n.d.	n.d.	n.d.	n.d.	41.5	n.d.	n.d.	n.d.	1131
	7	n.d.	n.d.	n.d.	n.d.	14.3	185	46.2	n.d.	33.3	1810
	8	n.d.	n.d.	n.d.	n.d.	n.d.	n.d.	n.d.	n.d.	n.d.	1470
	9	n.d.	n.d.	n.d.	n.d.	n.d.	41.5	n.d.	n.d.	n.d.	2941
	10	n.d.	n.d.	n.d.	n.d.	n.d.	n.d.	n.d.	n.d.	n.d.	249

Concentrations are reported as estimated neat oral fluid concentrations, assuming an oral fluid volume of 1 mL. M = morphine; C = codeine; M3G = morphine-3-glucuronide; M6G = morphine-6-glucuronide; C6G = codeine-6-glucuronide; crea = creatinine; n.d.= not detected.

Following the ingestion of raw poppy seeds, morphine was detected in all oral fluid samples at 0.5 hours, with a median morphine concentration in neat oral fluid of 3.3 (range 1.4–5.6) ng/mL. Codeine was also detected in three out of five oral fluid samples, with a median codeine concentration in neat oral fluid of 0.9 (range 0.8–1.1) ng/mL. After 2 hours, morphine was only detected in one of five oral fluid samples (neat oral fluid concentration 2.4 ng/mL), and codeine was not detected in any sample.

The consumption of crispbreads did not lead to detection of opioids in oral fluid samples at either of the two time points.

All five participants in the Raw seed group had at least one opioid, including opioid glucuronides, detected in urine. Three out of five participants in the Crispbread group had at least one opioid, including opioid glucuronides, detected in urine.

### Routine cases

We included 2253 oral fluid cases, of which the majority were from correctional facilities. In 1145 of the cases, morphine was detected, either alone (1002 cases) or combined with codeine (143 cases). In the remaining 1108 cases, only codeine was detected. The median concentration of morphine in neat oral fluid was 56 (range 2.6–154 322) ng/mL. For codeine, the median concentration was 43 (range 2.7–13 810) ng/mL. In the 1002 morphine only cases, the median concentration was 61 (range 2.6–15 881) ng/mL.

The concentrations in the routine cases were compared with the results from the experimental study. We excluded cases where codeine was detected above cut-off level (0.90 ng/mL, equivalent to 2.7 ng/mL in neat oral fluid) since none of the samples in the experimental study exceeded this threshold. The percentage of routine cases with morphine concentrations within the range (≤5.6 ng/mL, neat oral fluid) observed in the experimental study was calculated. These cases (*n* = 142, 14% of 1002) were considered consistent with the poppy seed defense, as determined by the present experimental study results.

## Discussion

This study shows that ingestion of raw poppy seeds with an opioid content well below the EU-regulated maximum levels resulted in the detection of opioids in oral fluid, as well as in urine. The detection window in oral fluid appeared short, as opioids were detected in all samples after 0.5 hours, but only in one of five samples 2 hours after consumption of the raw seeds. Consumption of crispbreads containing poppy seeds did not lead to detection of opioids in oral fluid, even though opioids were detected in urine in three out of five cases. Comparison with concentrations observed in real-life drug testing cases indicated that the poppy seed defense may be a challenge when interpreting oral fluid results, especially when low cut-off levels are applied.

The finding of a short detection window of opioids in oral fluid after consumption of raw poppy seeds is in accordance with two studies in which the opioid content of the poppy seeds was not measured [12, 13]. In another study, participants consumed a poppy seed roll or raw seeds, resulting in a dose of approximately 3 mg of morphine per serving [10], compared to only 0.1 mg morphine consumed through raw seeds in our study. Despite the high dose of morphine in that study, the detection window for opioids in oral fluid was three hours or less. However, the applied LOQ was also quite high (7.5 ng/mL) compared to the present study, which shortens the detection time. Our analytical method had a low LOQ (0.09 ng/mL for both morphine and codeine), but the consumed amount of opioids was also low. Two other studies found a longer detection window, up to approximately 24 hours for morphine and codeine in oral fluid, using an LOQ of 1 ng/mL [9, 11]. However, the total opioid dose administered in these two studies was very high; participants ingested two doses of raw poppy seeds eight hours apart, with each dose containing 15.7 mg morphine and 3.1 mg codeine.

Consumption of crispbreads did not lead to detection of opioids in oral fluid in our experimental study. These results are consistent with a study that did not detect opioids in oral fluid after the consumption of three bagels with poppy seeds [13]. The five crispbreads consumed in our study were estimated to contain 3–4 g of poppy seeds in total, but the opioid content was not measured. Processing poppy seeds has been shown to reduce their opioid content, and baking at high temperatures may reduce the opioid level to an especially large degree [1]. In contrast to our results, another study did detect morphine in oral fluid after consumption of baked goods, but the food contained significantly more poppy seeds than the crispbreads in our study [10]. Certain food products, like poppy seed cakes, can contain very high amounts of poppy seeds, typically from 10% to 30% [1]. However, in Scandinavia, crispbreads or bagels sprinkled with poppy seeds are more commonly consumed, making the intake of five crispbreads a more realistic scenario in our region.

We detected morphine more often than codeine in oral fluid after intake of raw poppy seeds. This was not surprising, since the ingested seeds contained more morphine than codeine. A previous study reported that levels of morphine were higher than those of codeine in all 83 poppy seed samples that were analyzed [1].

Opioids were detected in urine in all cases in the Raw seed group, and in three of five cases in the Crispbread group. Our findings are in accordance with several previous studies that have detected opioids in urine after intake of poppy seeds [4–8, 10, 13]. However, the current study was not designed to explore the detection window in urine in detail, since we only collected one urine sample two hours after ingestion of poppy seeds.

A relevant fraction (14%) of routine oral fluid samples contained morphine concentrations that were within the same concentration range as those observed in the current experiment following raw poppy seed consumption. These findings suggest that the poppy seed defense may be relevant in real-life oral fluid drug testing. However, it is important to note that the highest morphine concentrations in our experimental study were found only 0.5 hours after consumption of raw seeds, whereas only one sample had detectable levels of opioids two hours after consumption, indicating a short detection window. None of the morphine concentrations in our experimental study exceeded 15 ng/mL in neat oral fluid, which is the maximum confirmatory cut-off level recommended by the European Workplace Drug Testing Society (EWDTS) and the US Substance Abuse and Mental Health Services Administration (SAMHSA) [19, 20]. The LOQ in our experimental study, and the cut-off levels applied in our routine casework, are much lower than 15 ng/mL. Applying a low cut-off level increases the risk of detecting opioids from poppy seed ingestion, but also enhances the likelihood of identifying opioid use.

A strength of our experimental study is the use of commercially available raw poppy seeds. The quantity of raw seeds ingested (30 g) was somewhat higher than what we consider a typical dietary intake, but similar amounts may easily be incorporated in cakes, smoothies, or other raw food. Study participants were not instructed to rinse their mouths after consumption of the seeds. This could lead to poppy seed residues remaining in the mouth during oral fluid sampling, which is a possible scenario in real-life cases.

The small number of study participants is a limitation of our study. Additionally, the opioid content in the raw poppy seeds was substantially lower than the EU maximum levels. According to a report from the Norwegian Food Safety Authority [21], 19 of 20 tested poppy seed products contained opium alkaloids in 2023, with concentrations of morphine equivalents ranging from 1.4 to 131 mg/kg. The content of morphine equivalents in the poppy seeds used in the present experimental study was 3.2 mg/kg and therefore in the lower range. However, two separate batches of the same product brand were also tested by the Food Safety Authority, who found a substantially higher opioid content (morphine 15.3 mg/kg and 18.0 mg/kg, and codeine 1.6 and 1.5 mg/kg, respectively) than the present study [21]. This could be explained by variation in opioid content between batches, and even within a batch, but we cannot rule out that our seed sample preparation reduced the opioid content prior to analysis. We ground our seeds manually using a mortar. In contrast, the laboratory conducting the analyses for the Food Safety Authority dry-froze their seeds prior to pulverization (personal correspondence). However, we consider the exact opioid content of the poppy seeds to be of lesser importance, as the levels were below the EU legal limits in both analyses. Although the opioid content of the crispbreads was unknown, the detection of opioids in urine following their consumption suggests that the crispbreads contained opioids.

## Conclusion

Consumption of raw poppy seeds with low levels of opioids led to detection of opioids in oral fluid as well as urine, but the detection time in oral fluid appeared short. Consumption of crispbreads did not result in detection of opioids in oral fluid, but opioids were detected in urine. Comparison with routine drug testing cases indicated that the poppy seed defense may be a challenge when interpreting oral fluid results, particularly when low cut-off levels are applied. Therefore, when evaluating the poppy seed defense, it is important to consider the quantity of seeds consumed, the interval between ingestion and sample collection, as well as the laboratory’s cut-off level.

## Data Availability

Data from the experimental study are available in the article. Data from routine cases will be shared upon reasonable request to the corresponding author.
